# Optimization of Shikonin Homogenate Extraction from *Arnebia euchroma* Using Response Surface Methodology

**DOI:** 10.3390/molecules18010466

**Published:** 2013-01-02

**Authors:** Tingting Liu, Chunhui Ma, Lei Yang, Wenjie Wang, Xiaoyu Sui, Chunjian Zhao, Yuangang Zu

**Affiliations:** 1 Key Laboratory of Forest Plant Ecology, Ministry of Education, Northeast Forestry University, Harbin 150040, China; E-Mails: liutingting5@gmail.com (T.L.); ylmanefu@163.com (C.M.); wjwang225@hotmail.com (W.W.); zcjsj@163.com (C.Z.); 2 College of Pharmacy, Qiqihar Medical University, Qiqihar 161006, China; E-Mail: suixiaoyu@gmail.com

**Keywords:** *Arnebia euchroma*, shikonin, homogenate extraction, response surface methodology

## Abstract

An efficient homogenate extraction technique was employed for extracting shikonin from *Arnebia euchroma*. The homogenate extraction procedure was optimized and compared with other conventional extraction techniques. The proposed method gave the best result with the highest extraction efficiency in the shortest extraction time. Based on single-factor experiments, a three-factor-three-level experimental design has been developed by Box-Behnken design. The optimal conditions were 78% ethanol as solvent, homogenate extraction time of 4.2 min, 10.3 liquid to solid ratio and two extraction cycles. Moreover, the proposed method was validated by stability, repeatability and recovery experiments. The developed homogenate extraction method provided a good alternative for the extraction of shikonin from *A. euchroma*. The results indicated that the proposed homogenate extraction was a convenient, rapid and efficient sample preparation technique and was environmental friendly. Furthermore, homogenate extraction has superiority in the extraction of thermally sensitive compounds from plant matrices.

## 1. Introduction

**Figure 1:**
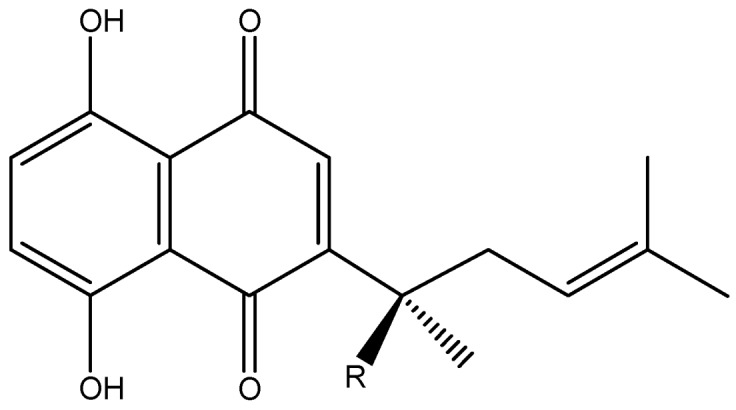
Structure of shikonin.

*Arnebia euchroma* is an important medicinal plant that grows mainly in the west and southwest China and has been used in complementary and alternative medicine for thousands of years [1]. Pharmacology studies indicated that *A. euchroma* has multiple pharmacological actions such as antioxidant [2], antimicrobial [3], antithrombotic [4], wound healing [5], anti-inflammatory [6,7] and anticancer activities [8,9]. To date, several chemical components that can be classified as naphthoquinones have been isolated from *A. euchroma*. Shikonin (Figure 1) is the major active constituent among these naphthoquinones [1]. 

For extract preparation, selection of an appropriate extraction method is a key consideration. In developed studies, many methods have been used for separation of shikonin from *A. euchroma*, such as supercritical fluid extraction [10,11,12], maceration extraction [13], heat reflux extraction [14], microwave-assisted extraction [15,16,17], ultrasound-assisted extraction [18,19], and so on. Supercritical CO_2_ extraction offers high selectivity, short operation time, high purity of the target ingredient, but its application is subject to the treatment capacity, so this method is accompanied by low yield and at present is difficult for industrial development. Traditionally, for the extraction of shikonin, maceration extraction using an organic solvent has been one of choices. These techniques are often time consuming and require large volumes of organic solvent, whose subsequent disposal creates severe environmental hazards. Shikonin is thermally unstable and could be degraded fast during the heat reflux and microwave-assisted extraction, if the temperature exceeds 60 °C [20,21]. Although many studies still use those two methods, spectrophotometry determination covered up this defect [14,15,16,17]. Ultrasonic extraction is a new technology for rapid extraction with high efficiency, but the problems including high energy consumption and noise pollution of industrial scale equipment are inevitable at present. As a result, the productivity of shikonin extraction from plants is very low, resulting in its extraordinarily high price [22].

Recently, the development and use of environmentally friendly methods has become increasingly popular. Homogenate extraction is an alternative to conventional extraction methods, through which chemical compositions are extracted from material into solvent by high-speed mechanical shearing, mixing, fluid cutting action and smashing without heating and pressure. Furthermore, homogenate extraction combines the comminution and extraction processes into one operation, avoiding dust pollution. The method has been documented to be effective in extracting alkaloids and terpene alcohols from leaves [23,24,25], and flavonoids from seeds and fruits [26,27,28]. However, its application on the extraction of shikonin from plant root has not been reported. 

The aim of this work was to develop a convenient, efficient, rapid and environmentally friendly homogenate approach for the extraction of shikonin from radix *A. euchroma*, and to compare the results with conventional extraction methods. It was found that parameters including the volume fraction of ethanol, liquid to solid ratio, and homogenate time were influential on the final yield and purity, and these parameters were optimized systematically.

## 2. Results and Discussion

### 2.1. Single-Factor Shikonin Extraction Experiments

The factors concerning homogenate extraction of shikonin included volume fraction of ethanol, homogenate extraction time, liquid to solid ratio and number of extraction cycles. The influence of each factor was studied by single-factor experiments. All assays were conducted in triplicate.

#### 2.1.1. Effect of Volume Fraction of Ethanol

The selection of a suitable solvent for extracting the target compounds from the plant matrix is a fundamental step. The extractions were carried out in aqueous ethanol solutions at different concentrations (volume fraction of ethanol ranging from 50% to 100%) with homogenate extraction time of 4 min, liquid to solid ratio 10 mL:1 g.

**Figure 2:**
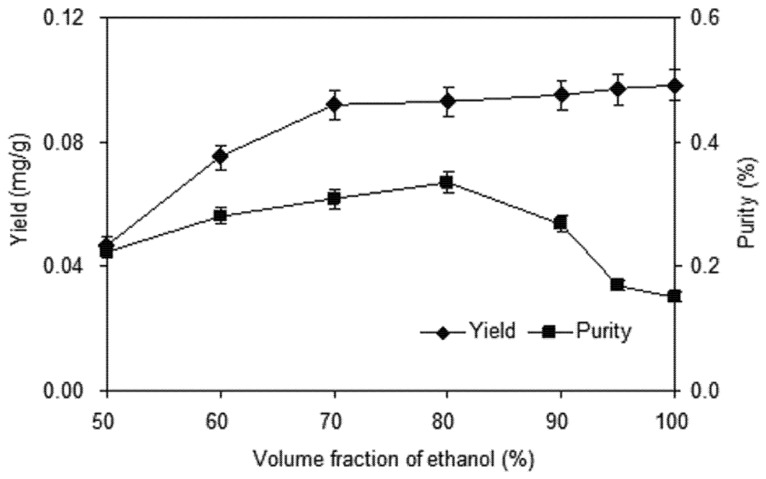
Effect of ethanol volume fraction on the yield and purity of shikonin.

Figure 2 provides the extraction yield and purity of shikonin from radix *A. euchroma* in these experiments. It can be observed that the extractions of shikonin from radix *A. euchroma* were greatly influenced by the ethanol concentration. The yields increased obviously with the increase of ethanol concentration up to 80%. When extracted with 90% ethanol solution, the extraction yields show a little increase. By contrast, the purity of shikonin increased when the volume fraction of ethanol in the range of 50%–80%. The decreasing extraction solvent polarity would bring the low-polarity chemical constituents such as β-sitosterol and benzoquinones into the solvent, which had an influence on the purity of shikonin in the extracts. Finally, a volume fraction of ethanol range of 70%–90% was adopted in further optimization studies.

#### 2.1.2. Effect of Homogenate Extraction Time

**Figure 3:**
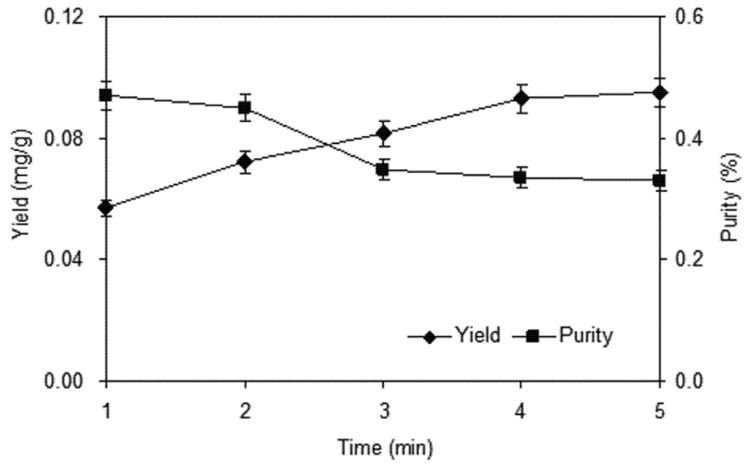
Effect of homogenate extraction time on the yield and purity of shikonin.

To select a proper homogenate time is to obtain complete extraction. Traditionally, higher yield requires a longer extraction period. To investigate the influence of homogenate extraction time on yield and purity of shikonin, a 10 g sample was extracted under the conditions of 100 mL of 80% ethanol for different extraction times (ranging from 1 to 5 min). The results shown in Figure 3 clearly indicate that when homogenate extraction time increased from 1 to 4 min, the yield of shikonin increased dramatically. When the time was longer than 4 min, the time effect was not significant. As for purity, as homogenate extraction time increased, however, a slight decrease was observed. In view of this result, a 3–5 min treatment time was selected for further optimization experiments.

#### 2.1.3. Effect of Liquid to Solid Ratio

The liquid to solid ratio is also an important factor in the extraction. In general, a higher solvent volume can dissolve the target compound more effectively and result in a better extraction yield.

**Figure 4:**
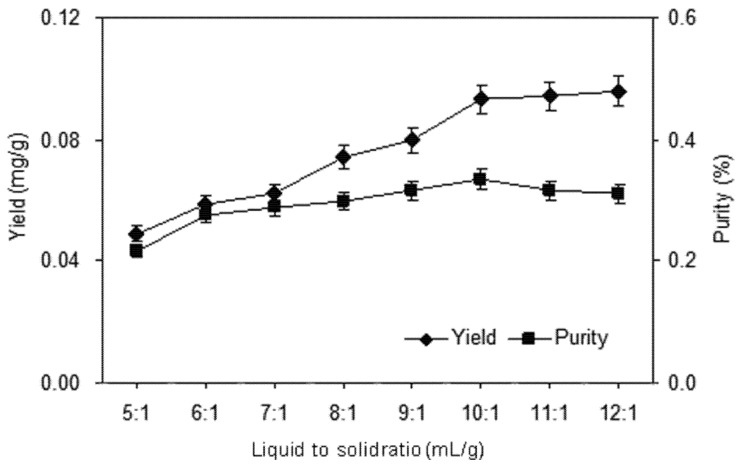
Effect of liquid to solid ratio on the yield and purity of shikonin.

Large solvent volumes could make the procedure difficult and lead to unnecessary waste, while small volumes may lead to incomplete extraction. A series of extractions were carried out with different liquid to solid ratios (5:1, 6:1, 7:1, 8:1, 9:1, 10:1, 11:1, and 12:1 mL/g) to evaluate the effect of the liquid to solid ratio. Results shown in Figure 4 indicated an obvious increase of yield and purity of shikonin before the liquid to solid ratio reached 10:1. When the liquid to solid ratio was increased from 10:1 to 12:1, however, the yield was not significantly improved and the purity was decreased slightly. Thus, a liquid to solid ratio range of 9:1–11:1 is used in the further optimization study.

#### 2.1.4. Effect of Number of Extraction Cycles

**Figure 5:**
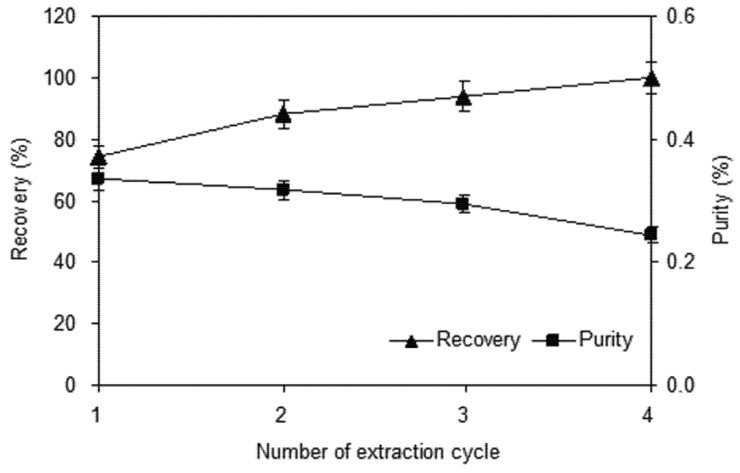
Effect of number of extraction cycles on the yield and purity of shikonin.

The effect of successive extractions of the residue on yield and purity was investigated. The solid residue was re-extracted using fresh ethanol solution each time. In this experiment, the effect of extraction cycle number on the extraction efficiency within the range of 1–4 was investigated. The recovery is expressed as the observed value of shikonin and the cumulative extraction yield of four times was taken to be 100%. It can be seen in Figure 5 that the recovery increased slowly with the number of extraction cycles, although only a small increase was observed after two cycles. The purity of shikonin decreased slowly when number of extraction cycles increased. To save solvent, energy and time, two cycle extraction is sufficient to ensure recovery of most of the shikonin content of the plant. In continuous mass production, two cycle extraction is suggested and the extracting solution in second cycle also can be used as solvent in the next production batch.

### 2.2. Parameter Optimization by Response Surface Methodology

#### 2.2.1. Model Building and Statistical Analysis

The experimental data obtained from the 17-run-experiment is given in Table 1. There were a total of 17 runs for optimizing the three individual parameters which were applied to the yield and purity of shikonin. Each run was carried out in triplicate, and the yields and purities of shikonin were the average of three sets of experiments. The results of each dependent variable with their coefficients of determination (*R*^2^) are summarized in Table 2. Statistical analysis indicated that the proposed model was adequate, possessing no significant lack of fit and with satisfactory values of the *R*^2^ for the yield and purity. The *R*^2^ values for the yield and purity were 0.985 and 0.963, respectively. The coefficients of variances for yield and purity were 2.52 and 3.63, respectively. In general, a high coefficient of variances indicates that variation in the mean value is high and does not satisfactorily develop an adequate response model [29]. The probability (*p*) values of both the regression models were less than 0.05. According to the model (Table 2), as for the yield, linear terms of homogenate time (*X_1_*, *p *< 0.01), liquid to solid ratio (*X_2_*, *p *< 0.0001) volume fraction of ethanol, (*X_3_*, *p *< 0.0001); the quadratic terms of homogenate time (*X_1_*^2^, *p* < 0.05), liquid to solid ratio (*X_2_*^2^, *p* < 0.0001), volume fraction of ethanol (*X_3_*^2^, *p *< 0.01) reached statistical significance. The results suggested that the change in the above three factors had a significant effect on the shikonin yield in the extracts. In contrast, the interactions between homogenate extraction time, liquid to solid ratio and volume fraction of ethanol were not statistically significant. The “Lack of Fit *F*-value” of 0.86 implied the Lack of Fit was significant. The probability for occurring of such a "Lack of Fit *F*-value" was only 0.52% and can be treated as statistical noise, indicating excellent agreement of the experiment values with the predicted values.

**Table 1:** Experimental data and the observed response value with different combinations of homogenate extraction time (*X_1_*), liquid to solid ratio (*X_2_*) and volume fraction of ethanol (*X_3_*) used in the Box-Behnken design.

Run No.	Experimental Design			Dependent Variables	
	*X_1_*: Homogenate time (min)	*X_2_*: Liquid to solid ratio (mL/g)	*X_3_*: Volume fraction of ethanol (%)	Yield of shikonin *Y* (mg/g)	Purity of shikonin *P* (%)
1	5	10	90	0.106	0.25
2	4	10	80	0.099	0.32
3	4	10	80	0.094	0.33
4	4	11	90	0.105	0.22
5	3	10	90	0.099	0.25
6	4	9	90	0.087	0.23
7	3	11	80	0.091	0.28
8	5	10	70	0.096	0.29
9	3	9	80	0.066	0.30
10	4	10	80	0.096	0.33
11	4	10	80	0.093	0.35
12	5	11	80	0.100	0.27
13	4	11	70	0.096	0.29
14	4	9	70	0.068	0.30
15	4	10	80	0.093	0.33
16	3	10	70	0.082	0.30
17	5	9	80	0.071	0.29

**Table 2:** Estimated regression coefficients for the quadratic polynomial model and ANOVA for the experimental results in the optimization of shikonin extractions.

Regression coefficients	Value	Sum of Squares	Degree of freedom	Mean Square	*F* value	Prob > *F*
Yield (mg/g)						
Model		24.06	9	2.67	51.19	<0.0001
*β_0_*	9.51					
*β_1_*	0.45	1.64	1	1.64	31.44	0.0008
*β_2_*	1.27	12.84	1	12.84	245.85	<0.0001
*β_3_*	0.69	3.79	1	3.79	72.57	<0.0001
*β_12_*	0.11	0.05	1	0.05	0.97	0.3569
*β_13_*	−0.18	0.13	1	0.13	2.55	0.1541
*β_23_*	−0.25	0.25	1	0.25	4.84	0.0638
*β_11_*	−0.33	0.46	1	0.46	8.72	0.0213
*β_22_*	−1.01	4.28	1	4.28	82.02	<0.0001
*β_33_*	0.40	0.68	1	0.68	13.02	0.0086
Lack of Fit		0.14	3	0.05	0.86	0.5294
Purity (%)						
Model		201.15	9	22.35	20.20	0.0003
*β_0_*	33.09					
*β_1_*	−0.54	2.30	1	2.30	2.08	0.1927
*β_2_*	−0.75	4.45	1	4.45	4.02	0.0849
*β_3_*	−2.89	66.75	1	66.75	60.31	0.0001
*β_12_*	0.02	0.001	1	0.001	0.001	0.9700
*β_13_*	0.27	0.30	1	0.30	0.27	0.6177
*β_23_*	−0.16	0.10	1	0.10	0.09	0.7750
*β_11_*	−1.80	13.65	1	13.65	12.33	0.0098
*β_22_*	−2.92	35.96	1	35.96	32.49	0.0007
*β_33_*	−3.95	65.72	1	65.72	59.38	0.0001
Lack of Fit		3.79	3	1.26	1.27	0.3963

#### 2.2.2. Interpretation of Response Surface Models

**Figure 6:**
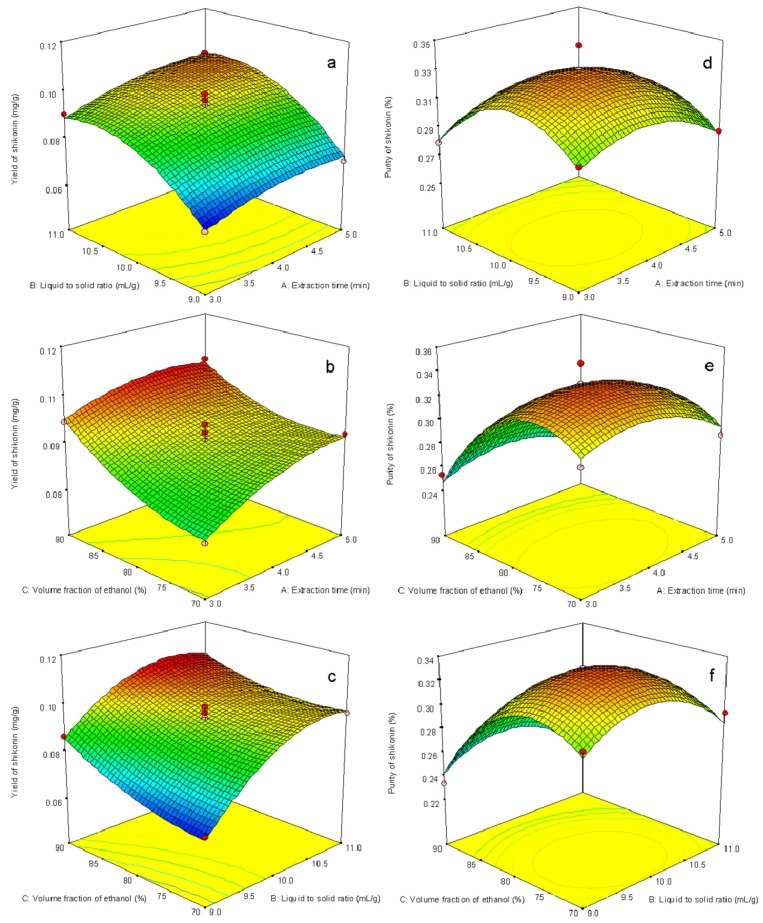
The 3D response surface of yield and purity affected by the varying homogenate extraction time and liquid to solid ratio (**a**, **d**), the varying homogenate extraction time and volume fraction of ethanol (**b**, **e**), and the varying liquid to solid ratio and volume fraction of ethanol (**c**, **f**). The values of the fixed parameters are as follows: (**a**, **d**): volume fraction of ethanol of 80%; (**b**, **e**): liquid to solid ratio of 10 mL/g; (**c**, **f**): homogenate extraction time of 4 min.

Response surfaces were plotted to study the effects of parameters and their interactions on extraction yield and purity. Three-dimensional response surface plots are presented in Figure 6. These types of plots show effects of two factors on the response at a time. Figure 6a is the response surface and contour plot showing the effect of homogenate extraction time and liquid to solid ratio on the response at the fixed ethanol concentration. Both extraction time and liquid to solid ratio have positive effects on the response. It can be seen that by increasing the extraction time the shikonin yields increased as well, reached a maximum value, and the further increase had slightly effect. The response increased rapidly with the increase of liquid to solid ratio and reached a maximum value, followed with equilibrium with its further increase. Figure 6b depicts the interaction effect of extraction time and volume fraction of ethanol on the yield at the fixed value of liquid to solid ratio. The increase of volume fraction of ethanol can significantly enhance the response, and then amaximum response was obtained. Figure 6c describes the interaction effect of liquid to solid ratio and volume fraction of ethanol on the yield response at the fixed value of extraction time.

#### 2.2.3. Verification Tests

The prediction optimization values were calculated using the second-order polynomial equation. The optimum conditions given by the model were as follows: 4.2 min homogenate extraction time at 78% volume fraction of ethanol and 10.3:1 liquid to solid ratio. Under these conditions, the model gave predicted values of extraction yield of being 0.097 mg/g and purity of 0.32%. To test the validity of the response surface analysis method, the extraction was carried out under the proposed conditions, the extraction yield and purity were 0.095 mg/g and 0.34%, respectively (n = 5). The good correlation between these results confirmed that the response model was adequate to reflect the expected optimization.

### 2.3. Method Validation

#### 2.3.1. Stability

The stability of shikonin in ethanol aqueous solution was evaluated by determining standard solution of shikonin after homogenate extraction and one week later. The recovery of shikonin was taken to evaluate their stability at the obtained operating extraction conditions. As shown in Table 3, the results indicated that complete recovery at the operating extraction conditions varied from 98.18% to 99.51% for shikonin with no change in retention time observed for the analyte. After one week, the recovery of shikonin was 87.77%–90.91%, this is due to the fact that quinone compound is susceptible to photooxidation by exposure to air and light in a storage period [4]. But the complete recovery after homogenate extraction indicated the rapid process could reduce the loss from photooxidation to a great extent.

**Table 3:** Stability studies of standards shikonin under optimum homogenate extraction conditions.

Concentration level	Initial concentration (μg/mL)	Recovered concentration after extraction (μg/mL)	RSD% (n = 3)	Average recovery (%)	Recovered concentration after 7 day (μg/mL)	RSD% (n = 3)	Average recovery (%)
1	0.08	0.08	0.37	99.51	0.07	0.64	87.77
2	0.55	0.54	0.33	98.18	0.50	0.55	90.91

#### 2.3.2. Recovery

To evaluate the accuracy of the proposed method, standard solution of shikonin were added to radix *A. euchroma* samples, at three levels, respectively. Under optimized conditions, samples with added standard solution were extracted by homogenate extraction, and then determined by HPLC to examine the recovery of the proposed method. As shown in Table 4, satisfactory results were found, with average recovery values of 98.67%.

#### 2.3.3. Repeatability

To determine the repeatability of the proposed extraction method five samples of the same weight were processed under the optimum extraction conditions. The mean extraction yields of shikonin obtained under the optimized conditions showed good reproducibility, with calculated RSD values of 1.3%. This shows that the proposed homogenate extraction method has an acceptable level of repeatability. The results suggested that shikonin were stable in the process and in the extracts. These method validation studies indicate that the proposed method is credible.

**Table 4:** The recovery of shikonin from radix *A. euchroma* (n = 3).

Sample	Shikonin content of the sample determined (μg)	Amount of added shikonin standard (μg)	Amount of the sample determined with added shikonin standard (μg )	Recovery (%)
1	97	100	195	98.98
2	97	300	390	98.23
3	97	450	531	98.88
Average				98.67

### 2.4. Comparison of Different Extraction Method

**Figure 7:**
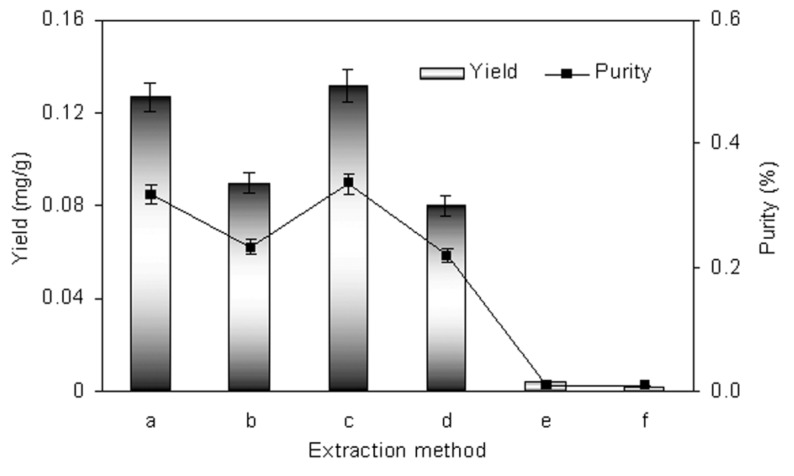
Comparison of homogenate extraction with other extraction methods, mean ± S.D. (n = 3). (**a**) homogenate extraction; (**b**) maceration extraction; (**c**) ultrasound extraction; (**d**) supercritical fluid CO**_2_** extraction; (**e**) microwave extraction; (**f**) hot reflux extraction.

**Figure 8:**
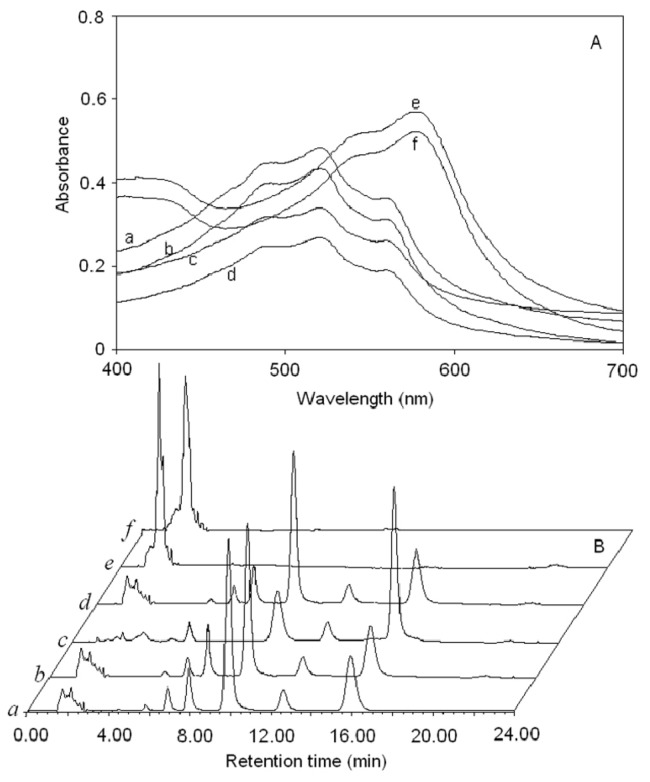
The visible spectra (**A**) and HPLC chromatograms (**B**) of samples extracts by different methods. (a) homogenate extraction; (b) ultrasonic extraction; (c) supercritical fluid CO_2_ extraction; (d) maceration extraction; (e) microwave extraction; (f) hot reflux extraction.

Yield and purity of shikonin by different extraction methods are shown in Figure 7. The visible spectra and HPLC chromatograms of *Arnebia euchroma *extracts by different extraction methods are provided in Figure 8. It can be observed the extraction yields of shikonin by homogenate and ultrasound extraction were higher than that obtained by other extraction methods. However, homogenate extraction possesses advantages compared to other methods for the extraction of compounds especially by saving in processing time, consuming less energy and achieving high extraction efficiency.

The extracts by microwave and hot reflux extraction were black purple, which was attributed to the high temperature in the system. As shown in Figure 8A e, f, visible spectra from microwave and hot reflux extraction were different from those obtained by other methods. Their absorption spectrum has obvious features such as the maximum absorption peak moved to 580 nm, while the authentic maximum absorption peak was 516 nm. This result suggested the shikonin and its ester derivatives have been destroyed. The maceration extraction and supercritical fluid CO_2_ extraction obtained good extracts, while their extraction completeness depended on a large extent on the time. 

From the HPLC chromatograms of samples (Figure 8B), ingredients of homogenate, ultrasound and maceration extracts were homologous, including shikonin and its five ester derivatives, which retention time were in the range of 5 to 18 min, but in the supercritical fluid CO_2_ extracts, there were only five chromatographic peaks in the range of 5 to 18 min, which indicated differences in the ingredient composition. Furthermore, no corresponding peaks were detected in the extract when microwave and hot reflux extraction were employed, which indicated that the composition of extracts changed dramatically.

These results demonstrate that the extraction efficiency of shikonin is dependent on the use of a normal temperature in the procedure. Homogenate extraction is developed as a potential and powerful alternative to conventional extraction techniques in the extraction of organic compounds from plant materials, especially for heat sensitive compounds.

## 3. Experimental

### 3.1. Materials and Reagents

Radix *A. euchroma* was purchased from Sankeshu Medicinal Materials Market, (Harbin, Heilongjiang Province, China) and identified by Prof. Shaoquan Nie from Key Laboratory of Forest Plant Ecology, Northeast Forestry University. The sample was preserved in a dry, cool environment, avoiding light exposure. Dried radix *A. euchroma* was preliminarily cut into 2- to 3-mm pieces with 8–10 mm diameter and stored in a refrigerator until use. The moisture of radix *A. euchroma* was 6.5%. Shikonin standard were purchased form National Institute for the Control of Pharmaceutical and Biological Products (Beijing, China). Methanol of chromatographic grade was purchased from J&K Chemical Ltd. (Beijing, China). Reverse osmosis Milli-Q water (Millipore, Bedford, MA, USA) was used for all solutions and dilutions. All of the solvents prepared for HPLC were filtered through 0.45 μm microporous membrane (Guangfu, Tianjin, China). Other reagents leveled in the analytical grade and were obtained from Beijing Chemical Reagents Co. (Beijing, China).

### 3.2. Apparatus

The extraction procedure was carried out in a Waring blender (Philips, Guangdong, China). The bath was a cylinder container (diam, 4.5 cm). The reamer rating was 10,000 rpm. The chromatographic system (Waters, Milford, MA, USA) consisted of Millennium 32 system software, 717 plus autosampler, 1525 pump, 717 automatic column temperature control box and 2487 UV detector. Chromatographic separation was performed on a Kromasil-C18 reversed-phase column (4.6 mm × 250 mm, 5 µm, Akzo Nobel Inc., Bohus, Sweden). 

### 3.3. HPLC Quantitative Analysis

For HPLC analysis, methanol–water (85:15, v/v) was used as the mobile phase. The mobile phase was filtered through a 0.45 µm membrane filter (Guangfu Chemical Reagents Co., Tianjin, China) and then deaerated ultrasonically prior to use. Shikonin was quantified by a UV detector at the wavelength of 516 nm following HPLC separation. Flow rate was 1.0 mL/min, the injection volume was 10 µL and the column temperature was maintained at 25 °C. The chromatographic peak of the analyte was confirmed by comparing its retention time with the reference standard. Quantification was carried out by the integration of the peak using external standard method. The working calibration curve based on shikonin standard solutions showed good linearity over the range of 0.8–66.9 µg/mL. The regression line for shikonin was *y* = 12788109 *x* − 6461 (*R* = 0.9997, *n* = 8), where *y* is the peak area of shikonin, and *x* is the concentration (µg/mL).

### 3.4. Spectrophotometric Qualitative Analysis

Absorbance of extraction solutions was diluted appropriately and scanned by spectrophotometer (UV-2550, Shimadzu, Kyoto, Japan) from 400–700 nm (reference: 80% ethanol).

### 3.5. Extraction Efficiency of Shikonin Evaluation

Selection of the most favorable extraction conditions was based on yield and purity of shikonin. After each extraction, the extract volume was measured, and 1 mL extract was diluted to a suitable concentration for the HPLC detection. Equation (1) was used to quantify yield of shikonin. The remaining extract solution was concentrated under vacuum to dryness at 55 °C and weighed. Equation (2) was used to quantify the purity of shikonin:

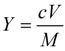
(1)

Equation (2) was used to quantify the purity of shikonin:

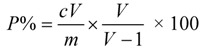
(2)
where *Y* is yield of shikonin (mg/g); *P * is purity of shikonin (%); *c* is concentration of shikonin (mg/mL); *V* is volume of extract solution (mL); *M* is mass of radix *A. euchroma *(mg); and *m* is mass of extract (mg).

### 3.6. Homogenate Extraction Process

The extraction of shikonin was performed by adding 10 g of sample powder into 100 mL of the selected volume fraction of ethanol in the Waring blender. After homogenate treatment, the extracts obtained were filtrated through a microporous membrane prior to HPLC analysis. The optimum volume fraction of ethanol, homogenate extraction time, liquid to solid ratio and number of extraction cycle, the yield and purity of shikonin as the response were systematically studied in this work.

### 3.7. Optimization Homogenate Extraction by Response Surface Methodology (RSM)

Response surface methodology was used to identify optimum parameters of three variables: volume fraction of ethanol, homogenate extraction time and liquid to solid ratio, regarding of two responses-yield and purity of shikonin. To estimate the model coefficients, a three-factor-three-level Box–Behnken design was carried out (17 runs). Independent variables (homogenate extraction time, liquid to solid ratio and volume fraction of ethanol) were standardized to the interval. The order of the experiments was randomized. A second-order polynomial equation was used to express the responses as a function of the independent variables:

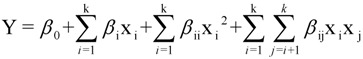
(3)
where *Y* is the predicted response, k the number of independent variables (factors), *x_i_* and *x_j_* the coded independent variables, *β_0_* the constant coefficient, and *β_i_*, *β_ii_* and *β_ij_* the coefficients of linear, quadratic and interaction term, respectively. Analysis of the experimental design data and calculation of predicted responses were carried out using Design Expert software (Trial Version 7.0.3, Stat-Ease Inc., Minneapolis, MN, USA). Additional confirmation experiments were subsequently conducted to verify the validity of the statistical experimental design.

### 3.8. Stability, Recovery and Repeatability of Homogenate Extraction

Stability tests was performed using shikonin standard dissolved in 78% volume fraction of ethanol by homogenate extraction under the optimum conditions (4.3 min homogenate time and liquid to solid ratio 10.3:1). The recovery of shikonin was taken as the indicative marker for the stability of shikonin at the derived operating extraction conditions. To determine the repeatability of the novel extraction method, five samples of the same weight (10 g) were processed under the same optimum extraction conditions as those obtained from the systematic study of different extraction parameters.

### 3.9. Reference Extraction Procedure

Powdered radix *A. euchroma* was weighed (10 g) and extracted by maceration, ultrasound, hot reflux, microwave and supercritical fluid CO_2_ extraction according to the appropriate references [11,13,14,16,19], respectively. The extract was filtrated through a 0.45 μm microporous membrane for HPLC and spectrophotometric analysis. 

### 3.10. Statistical Analysis

One way of ANOVA testing was used to calculate the significance of the differences of the extraction yield of shikonin and the purity of shikonin. The results were expressed as means of extraction yield ± the standard deviation (SD) and means of purity ± SD.

## 4. Conclusions

An efficient method has been developed for the extraction of shikonin from *A. euchroma*. The optimum conditions for homogenate extraction were studied. Under the optimized conditions, satisfactory extraction yield and purity of shikonin were obtained. Compared to other methods, the proposed approach provides higher extraction yield, purity of shikonin and significantly reduced extraction time, while avoiding dust pollution and use of a low temperature are recommendable. Homogenate extraction offered short extraction times and remarkable efficiency. Due to its good repeatability and precision, the proposed green and effective homogenate method shows great promise in the extraction and the separation of natural products.
